# A Gene Expression Signature Predicts Survival of Patients with Stage I Non-Small Cell Lung Cancer

**DOI:** 10.1371/journal.pmed.0030467

**Published:** 2006-12-26

**Authors:** Yan Lu, William Lemon, Peng-Yuan Liu, Yijun Yi, Carl Morrison, Ping Yang, Zhifu Sun, Janos Szoke, William L Gerald, Mark Watson, Ramaswamy Govindan, Ming You

**Affiliations:** 1 Department of Surgery, Washington University School of Medicine, St. Louis, Missouri, United States of America; 2 The Alvin J. Siteman Cancer Center, Washington University School of Medicine, St. Louis, Missouri, United States of America; 3 Department of Pathology, The Ohio State University Comprehensive Cancer Center, Columbus, Ohio, United States of America; 4 Department of Health Sciences Research, Mayo Clinic, Rochester, Minnesota, United States of America; 5 Department of Pathology, Memorial Sloan-Kettering Cancer Center, New York, New York, United States of America; 6 Department of Pathology and Immunology, Washington University in St. Louis, St. Louis, Missouri, United States of America; 7 Department of Internal Medicine, Washington University in St. Louis, St. Louis, Missouri, United States of America; University of Pittsburgh School of Medicine, United States of America

## Abstract

**Background:**

Lung cancer is the leading cause of cancer-related death in the United States. Nearly 50% of patients with stages I and II non-small cell lung cancer (NSCLC) will die from recurrent disease despite surgical resection. No reliable clinical or molecular predictors are currently available for identifying those at high risk for developing recurrent disease. As a consequence, it is not possible to select those high-risk patients for more aggressive therapies and assign less aggressive treatments to patients at low risk for recurrence.

**Methods and Findings:**

In this study, we applied a meta-analysis of datasets from seven different microarray studies on NSCLC for differentially expressed genes related to survival time (under 2 y and over 5 y). A consensus set of 4,905 genes from these studies was selected, and systematic bias adjustment in the datasets was performed by distance-weighted discrimination (DWD). We identified a gene expression signature consisting of 64 genes that is highly predictive of which stage I lung cancer patients may benefit from more aggressive therapy. Kaplan-Meier analysis of the overall survival of stage I NSCLC patients with the 64-gene expression signature demonstrated that the high- and low-risk groups are significantly different in their overall survival. Of the 64 genes, 11 are related to cancer metastasis *(APC, CDH8, IL8RB, LY6D, PCDHGA12, DSP, NID, ENPP2, CCR2, CASP8,* and *CASP10)* and eight are involved in apoptosis *(CASP8, CASP10, PIK3R1, BCL2, SON, INHA, PSEN1,* and *BIK)*.

**Conclusions:**

Our results indicate that gene expression signatures from several datasets can be reconciled. The resulting signature is useful in predicting survival of stage I NSCLC and might be useful in informing treatment decisions.

## Abstract 2

Meta-analysis of several lung cancer gene expression studies yields a set of 64 genes whose expression profile is useful in predicting survival of patients with early-stage lung cancer and possibly informing treatment decisions.

## Editors' Summary

**Background.:**

Lung cancer is the commonest cause of cancer-related death worldwide. Most cases are of a type called non-small cell lung cancer (NSCLC) and are mainly caused by smoking. Like other cancers, how NSCLC is treated depends on the “stage” at which it is detected. Stage IA NSCLCs are small and confined to the lung and can be removed surgically; patients with slightly larger stage IB tumors often receive chemotherapy after surgery. In stage II NSCLC, cancer cells may be present in lymph nodes near the tumor. Surgery plus chemotherapy is the usual treatment for this stage and for some stage III NSCLCs. However, in this stage, the tumor can be present throughout the chest and surgery is not always possible. For such cases and in stage IV NSCLC, where the tumor has spread throughout the body, patients are treated with chemotherapy alone. The stage at which NSCLC is detected also determines how well patients respond to treatment. Those who can be treated surgically do much better than those who can't. So, whereas only 2% of patients with stage IV lung cancer survive for 5 years after diagnosis, about 70% of patients with stage I or II lung cancer live at least this long.

**Why Was This Study Done?:**

Even stage I and II lung cancers often recur and there is no accurate way to identify the patients in which this will happen. If there was, these patients could be given aggressive chemotherapy, so the search is on for a “molecular signature” to help identify which NSCLCs are likely to recur. Unlike normal cells, cancer cells divide uncontrollably and can move around the body. These behavioral differences are caused by changes in their genetic material that alter their patterns of RNA transcription and protein expression. In this study, the researchers have investigated whether data from several microarray studies (a technique used to catalog the genes expressed in cells) can be pooled to construct a gene expression signature that predicts the survival of patients with stage I NSCLC.

**What Did the Researchers Do and Find?:**

The researchers took the data from seven independent microarray studies (including a new study of their own) that recorded gene expression profiles related to survival time (less than 2 years and greater than 5 years) for stage I NSCLC. Because these studies had been done in different places with slightly different techniques, the researchers applied a statistical tool called distance-weighted discrimination to smooth out any systematic differences among the studies before identifying 64 genes whose expression was associated with survival. Most of these genes are involved in cell adhesion, cell motility, cell proliferation, and cell death, all processes that are altered in cancer cells. The researchers then developed a statistical model that allowed them to use the gene expression and survival data to calculate risk scores for nearly 200 patients in five of the datasets. When they separated the patients into high and low risk groups on the basis of these scores, the two groups were significantly different in terms of survival time. Indeed, the gene expression signature was better at predicting outcome than routine staging. Finally, the researchers validated the gene expression signature by showing that it predicted survival with more than 85% accuracy in two independent datasets.

**What Do These Findings Mean?:**

The 64 gene expression signature identified here could help clinicians prepare treatment plans for patients with stage I NSCLC. Because it accurately predicts survival in patients with adenocarcinoma or squamous cell cancer (the two major subtypes of NSCLC), it potentially indicates which of these patients should receive aggressive chemotherapy and which can be spared this unpleasant treatment. Previous attempts to establish gene expression signatures to predict outcome have used data from small groups of patients and have failed when tested in additional patients. In contrast, this new signature seems to be generalizable. Nevertheless, its ability to predict outcomes must be confirmed in further studies before it is routinely adopted by oncologists for treatment planning.

**Additional Information.:**

Please access these Web sites via the online version of this summary at http://dx.doi.org/10.1371/journal.pmed.0030467.

- US National Cancer Institute information on lung cancer for patients and health professionals.
- MedlinePlus encyclopedia entries on small-cell and non-small-cell lung cancer.
- Cancer Research UK, information on patients about all aspects of lung cancer.
- Wikipedia pages on DNA microarrays and expression profiling (note that Wikipedia is a free online encyclopedia that anyone can edit).

## Introduction

Lung cancer is the leading cause of cancer death for both men and women in the US [1]. The high mortality among patients with lung cancer is mainly due to the absence of an effective screening strategy to identify lung cancer at an early stage [2]. Thus, only ~25% of patients presenting with lung cancer are in a sufficiently early stage to be amenable to effective surgical treatment. Patients with stage I or II non-small cell lung cancer (NSCLC) have ~70% five-year survival after surgery alone compared to less than a 5% five-year survival for advanced lung cancer (stages IIIB and IV) [3]. Even with surgical resection, almost half of those with stage I or II disease eventually die from recurrences.

Treatment choices for patients with NSCLC depend on the stage at which the cancer is diagnosed. Patients diagnosed with stage I NSCLC usually receive surgical resection only [4]. Patients with stage IA (T1N0M0) undergo resection and are rarely treated with adjuvant chemotherapy. Patients with resected stage IB–III (any T any N M0 except T1N0M0) NSCLC show improved survival when given adjuvant chemotherapy [4].

No reliable clinical or molecular predictors of recurrent disease are currently available. Because of heterogeneity in recurrence rates among patients with the same stage of cancer, it is critical to isolate a reliable molecular signature in tumors that could be used to identify those who are likely to develop recurrent disease and would thus benefit from adjuvant therapy. Moreover, identification of genes and molecular pathways critical for development of metastasis could lead to advances in therapeutics.

Several studies based on microarray technology have been performed to determine genetic profiles predictive of survival in NSCLC and to develop genomic approaches for stratifying risk [5–8]. However, the identified survival-related genes lacked consistency among these studies, likely due to limited patient samples, disease heterogeneity, and/or technical factors such as differences in microarray platforms and specimen processing. In this study, we conducted a meta-analysis of seven datasets to search for differentially expressed genes related to survival time (under 2 years, i.e., short-term survival and over 5 years, i.e., long-term survival). The data analyzed include our own previously unpublished dataset.

## Methods

### Data Collection

#### Samples from Washington University.

Thirty-six patients who underwent resection of stage IB NSCLC at Washington University School of Medicine (WUSM; St. Louis, Missouri, United States) were recruited for this study. These samples are referred to as dataset 1. Informed consent was obtained from the patients for tissue procurement prior to surgery and their medical records were maintained according to institutional guidelines and in conformance with HIPPA regulations. The overall survival data on all patients were censored on the date of the last follow-up visit or death from causes other than lung cancer. Tumor tissues were processed by the Human Tissue Bank and the Gene Chip Facility at WUSM according to standard operating procedures and protocols.

Briefly, frozen tissue samples at −80°C were pulverized and total cellular RNA was collected from each flash-frozen sample using TRIzol RNA isolation reagent (Invitrogen [http://www.invitrogen.com]). Total RNA was processed with a Qiagen (http://www.qiagen.com) RNeasy Mini kit. In vitro transcription-based RNA amplification was then performed on at least 8 μg of total RNA from each sample. Complementary DNA was synthesized using the T7-(dT)24 primer: 5′-GGCCAGTGAATTGTAATACGACT-CACTATAGGGAGGCGG-(dT)24–3′. The cDNA was processed using phase-lock gel (Fisher [http://www.fishersci.com; #E0032005101]) phenol/chloroform extraction. Next, in vitro transcriptional labeling with biotin was performed using the Enzo Bioarray Kit (Affymetrix [http://www.affymetrix.com; #900182]). The resulting cRNA was processed again using the Qiagen RNeasy Mini kit. Labeled cRNA was hybridized to HG_U95Av2 (Affymetrix) arrays according to manufacturer's instructions.

The raw fluorescence intensity data within CEL files were preprocessed with Robust Multichip Average (RMA) algorithm [9], as implemented with R packages from Bioconductor (http://www.bioconductor.org). This algorithm analyzes the microarray data in three steps: a background adjustment, quantile normalization, and finally summation of the probe intensities for each probe set using a log scale linear additive model for the log transform of (background corrected, normalized) PM intensities.

#### Samples from Mayo Clinic.

Eighteen patients with stage I squamous cell carcinoma (SCC) were selected from the patients diagnosed with lung cancer from 1997 to 2001 who underwent resection at Mayo Clinic, Rochester, Minnesota, United States. These samples are referred to as dataset 2. All enrolled patients and use of their tissues in the study were approved by the institutional review board of Mayo Clinic. The resected tumors were flash-frozen to −80 °C within 30 min after the tissues were surgically removed. The RNA isolation, cRNA synthesis, and microarray hybridization were performed as described by Sun et al. [6]. The raw fluorescence intensity data within CEL files were also preprocessed with the RMA algorithm.

#### Samples from other groups.

Dataset 3 was from Beer et al. [5] and includes 67 stage I primary lung adenocarcinomas (ADCs) (http://dot.ped.med.umich.edu:2000/ourimage/pub/Lung/index.html). Dataset 4 was from Bhattacharjee et al. [10] and includes 72 stage I lung ADCs (http://www.broad.mit.edu/mpr/lung/). Dataset 5 was from Borczuk et al. [8] and includes one squamous and three ADCs (http://hora.cpmc.columbia.edu/dept/pulmonary/5ResearchPages/Laboratories/powell%20supp1.htm). Dataset 6 was from Gerald et al. (unpublished data) and includes 63 stage I lung ADCs. Dataset 7 was from Bild et al. [11] and includes 33 squamous cell carcinomas and 31 ADCs (GEO accession number GSE3141). The raw data within the CEL files of these datasets were also preprocessed with the RMA algorithm.

All the samples used in our data analyses are listed in Table S1. Details of the clinical information for the subjects in each dataset are described in Table 1.

**Table 1:**
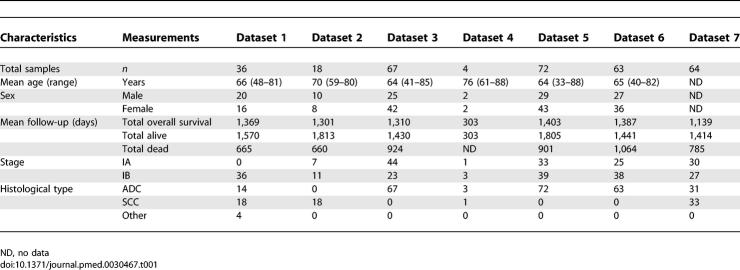
Clinical Summary of Patients in the Analyzed Datasets

### Data Processing

#### Gene matching.

Because several different microarray platforms were used in these datasets, the probe sets should be matched to identical genes. The batch query tool provided by Affymetrix (https://www.affymetrix.com/analysis/netaffx/batch_query.affx) was used for matching probe sets among datasets 1 to 7 [12]. Based on the latest UniGene clusters annotation provided by the manufacturer (NCBI Build 35.1), there were a total of 4,905 genes on all the five Affymetrix microarray systems HG_U95Av2, Hu6800, Hu133A, HG_U133AB, and Hu133plus2.

#### Distance-weighted discrimination.

Systematic differences from different datasets were remarkable, which would compromise the integrity of the data from different laboratories. To integrate the gene expression data from datasets 1 to 5, the distance-weighted discrimination (DWD) method (https://genome.unc.edu/pubsup/dwd/index.html) [13] was used to identify and adjust systematic differences that were present within these microarray datasets. The DWD method corrects for systematic biases across microarray batches by finding a separating hyperplane between the two batches and adjusting the data by projecting the different batches on the DWD plane, finding the batch mean, and then subtracting out the DWD plane multiplied by this mean [13]. All of the 197 samples from the five datasets were broken into two sub-branches, each of which was composed of samples from all of the five datasets (Figure S1). Poolability tests were performed to examine if these DWD-transformed gene expression data from different resources were poolable [14]. We randomly reshuffled data resources and generated 100 replicates of simulated data. We then compared the number of *p*-values below certain thresholds with the expected counts obtained by simulations that take into account the distributions of the DWD-transformed gene expression data and sample size in our study.

### Data Analysis

To preselect survival-related genes, ANOVA analysis was applied to 88 patients in datasets 1 to 5 who died within 2 years or survived beyond 5 years after surgery. Empirical *p*-values for each gene were obtained through 10,000 permutation tests. Genes with significant survival effects (*p* < 0.01) were selected for Cox proportional hazards regression analyses. Multivariate Cox proportional hazards regression analyses (adjusted for age, gender, cancer subtype, and cancer stage) with 10,000 bootstrap resampling were performed for each survival-related gene using all of 197 samples in datasets 1 to 5. The proportional hazards assumption for variables such as age, sex, cancer subtype, and cancer stage was investigated by examining the scaled Schoenfeld residuals. Sex and cancer stage generally displayed a significant deviation from this assumption. Therefore, these two variables were taken as strata and others as covariates in our Cox proportional hazards model. The plot of global *p*-values obtained by testing the proportional hazards assumption for all survival-related genes showed that the model used in our survival analysis was statistically warranted (Figure S2). The genes were ranked according to the bootstrap frequencies of *p* < 0.01 for their expression in regression models.

To identify a gene signature predictive of survival outcome, survival analyses were performed on all 197 samples in datasets 1 to 5. Partial Cox regression was performed to construct predictive components, and time-dependent ROC curve analysis was applied to evaluate the results [15]. The risk scores were calculated by a linear combination of the gene expression values for the selected genes, weighted by their estimated regression coefficients. All the samples were classified into high or low risk groups according to the risk scores. To choose an appropriate subset of genes for a common signature, we performed a forward selection procedure: (1) increase one gene each time based on the rank of genes that were identified in the above bootstrap analyses; (2) perform the partial Cox regression analysis and obtain the prediction accuracy using the chosen subset of genes; and (3) repeat steps 1 and 2 until the prediction accuracy is maximized. Kaplan-Meier survival plots, Mantel-Haenszel log rank tests, and time-dependent ROC analysis were implemented to assess the classification models according to the risk scores.

Hierarchical clustering based on a centered Pearson correlation coefficient algorithm and an average linkage method were used to show the expression patterns of survival-related genes in datasets 1 to 5.

All of the data analyses were implemented using the R statistical package [16]. A more detailed description of the data analyses is provided (Protocol S1).

### Quantitative RT-PCR Analysis

Using the samples from dataset 1, the relative expressions of nine randomly selected genes associated with survival were determined by quantitative RT-PCR (QRT-PCR) analysis as described in a previous report [17]. Primers for the QRT-PCR analysis (Table S2) were designed using Primer Express software version 2.0 (Applied Biosystems [http://www.appliedbiosystems.com]). Amplification of each target DNA was performed with SYBR Green master mix in Bio-Rad (http://www.bio-rad.com) Single Color Real-Time PCR Detection System according to the protocols provided. The control gene *β-actin* and the target genes amplified with equal efficiencies. To assess whether two amplicons have the same efficiency, the variation of ΔC_T_ (C_T,target_ – C_T,β-actin_, where C_T_ is cycle number at which the fluorescence signal exceeds background) with template dilution was evaluated [18]. The fold change of gene expression in long-term survival patients relative to short-term survival patients was calculated as 2^–ΔΔCT^ (ΔΔC_T_ = ΔC_T long_ – ΔC_T__short_). ANOVA was performed to determine differences among the groups. A *p*-value of less than 0.05 was considered to indicate statistical significance.

### Tissue Microarray

Lung tissues of 60 stage I NSCLC patients (including 12 patients dead by 2 years after surgical resection and 48 alive for more than 5 years) were collected during surgery between 1985 and 1999 at the Arthur James Cancer Hospital of the Ohio State University Medical School (Columbus, Ohio, United States). All tissues were fixed in formalin and embedded in paraffin. A patient tissue microarray was constructed from these tissues for examination of CRABP1 and ABCC1 immunoreactivity in short- and long-term survival patients. All antibodies were antigen-retrieved in a vegetable steamer with TRS, pH 6.1 (Dako [http://www.dako.com]), staining was performed on a Dako autostainer, and all primary incubations were for 1 h at room temperature. For CRABP1 (Abcam [http://www.abcam.com, #Ab2816], dilution 1:1000), detection kit used was LSAB+ (Dako). For ABCC1 (AXXORA [http://www.axxora.com, ALX-801–007-C125], dilution 1:50), the detection kit used was Vectastain Elite (Vector Labs [http://www.vectorlabs.com]). The immunohistochemical staining images were scanned using an ImageScope (Aperio [http://www.aperio.com]). The percentage of positive cancer cells was scored on a semiquantitative scale as 0 (0%), 1 (1%–25%), 2 (25%–50%), 3 (50%–75%), and 4 (75%–100%). Intensity was scored as 1 (weak), 2 (intermediate), and 3 (strong). Results were calculated by multiplying the score of percentage of positive cells (P) by the intensity (I). The maximum score was 12. Two investigators did the evaluation of immunostaining results independently. Student's t-test was used in statistical analyses.

## Results

### Differentially Expressed Genes Associated with Survival

Tables 2 and S3 list the genes related to overall survival in the combined data (*p* < 0.01). As shown in Table 2, we observed relatively consistent changes for both genes whose expression in low-risk patients is higher than in high-risk patients and genes whose expression in high-risk patients is higher than in low-risk patients. Since we did not use data from normal paired lungs in these analyses, it is not clear whether these genes are all overexpressed in both low-risk and high-risk patients. Therefore, there are at least four possibilities of gene-expression patterns: (1) one group of genes overexpressed in low-risk patients and another group of genes overexpressed in high-risk patients; (2) one group of genes overexpressed in high-risk patients and another group of genes underexpressed in high-risk patients; (3) one group of genes overexpressed in low-risk patients and another group of genes underexpressed in low-risk patients; or (4) a mixture of all three scenarios. In order to clarify this issue, we have begun to systematically acquire microarray information from all paired normal lungs in an attempt to determine possible expression patterns of these survival-related genes by comparing normal gene expression levels between low-risk and high-risk patients. The results from this ongoing study will be included a future publication.

**Table 2:**
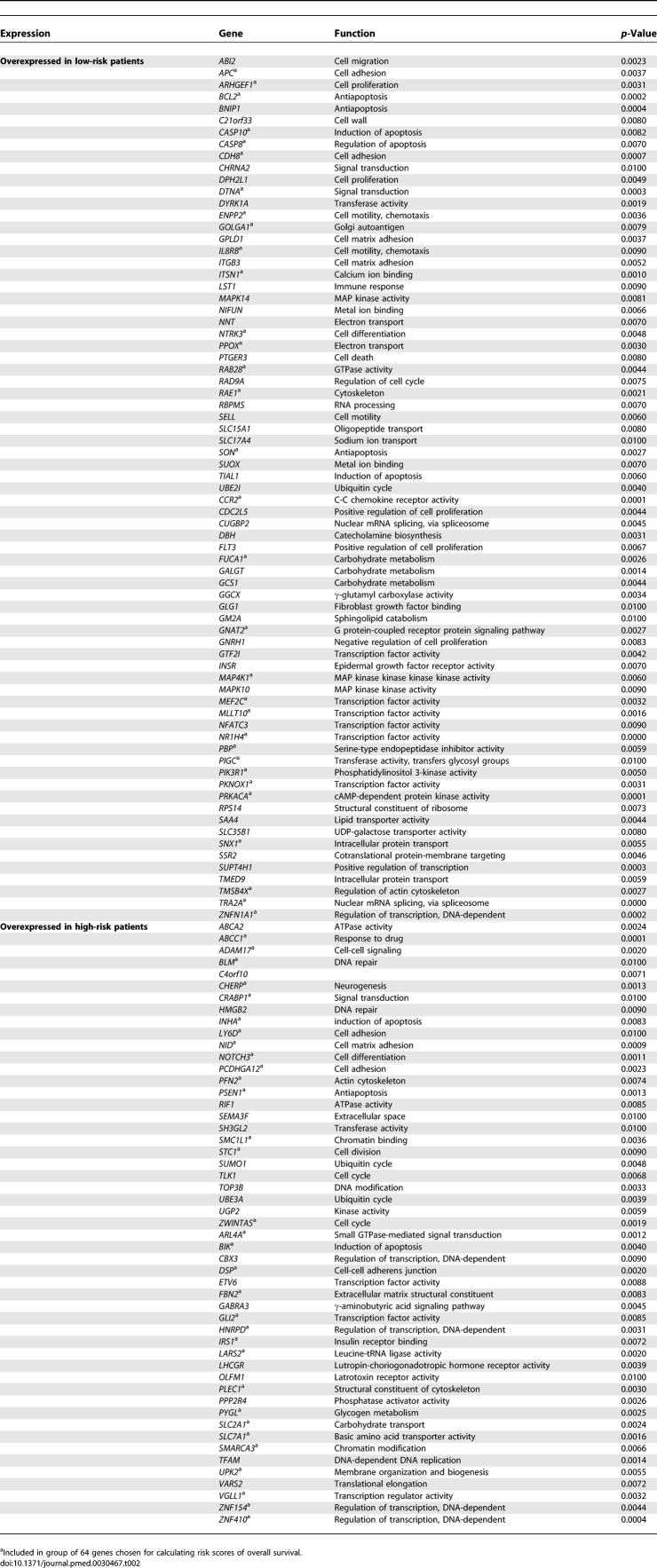
Genes Related to Survival

Most of these genes are related to cell adhesion, cell motility, cell proliferation, and apoptosis. Notably, several genes have been reported to be associated with cancer survival *(APC, IRS1, SLC2A1, BCL2, ABCC1, FLT3, RAD9A, Inhibin A, NTRK3, CASP8,* and *CASP10).* The *APC* gene plays a role in NSCLC, and high *APC* promoter methylation is significantly associated with poor survival in NSCLC [19]. *IRS1* is a high-risk classifier gene associated with cancer death within 12 months [8]. In another study, *BCL2* was observed to be up-regulated in a group of long-term survival patients with NSCLC [20]. *ABCC1* expression levels have been shown to be an independent predictor for disease-free survival in adult acute myeloid leukemia [21]. Acute myeloid leukemia patients with *FLT3* mutations tend to have poor prognoses [22]. In addition, *RAD9A*, which is involved in DNA repair, was found to be increased in radioresistant cells over radiosensitive cells [23]. *Inhibin A* was found to be overexpressed in two cases of primary clear cell renal cell carcinoma (2/16 [13%]) and three cases of metastatic clear cell renal cell carcinoma (3/5 [60%]) [24]. The expression of CASP8 and CASP10 was frequently decreased at the mRNA and protein levels in lung cancer progression [25]. We found that the genes encoding these two caspases were up-regulated in long-term survival patients. High *NTRK3* mRNA expression generally presages longer survival [26]. Four survival genes *(TMSB4X, INHA, FUCA1,* and *STC1)* were previously identified by the cross-validation procedure in dataset 3 [5]. Not surprisingly, several genes were reported to be involved in cancer progression, survival, or cancer subtypes in the original reports. For example, *ATP2B1, AKAP12, TNFAIP6, RGS16, HSPA8, RPS3, ADM,* and *P2RX5* are survival-associated genes [8]. *MUC1* may play a role in progression and invasiveness of colorectal carcinomas [27]. Finally *AGT, XBP1,* and *PODXL* are overexpressed in ADC compared with SCC [28].

### Validation of Selected Genes

**Figure 1:**
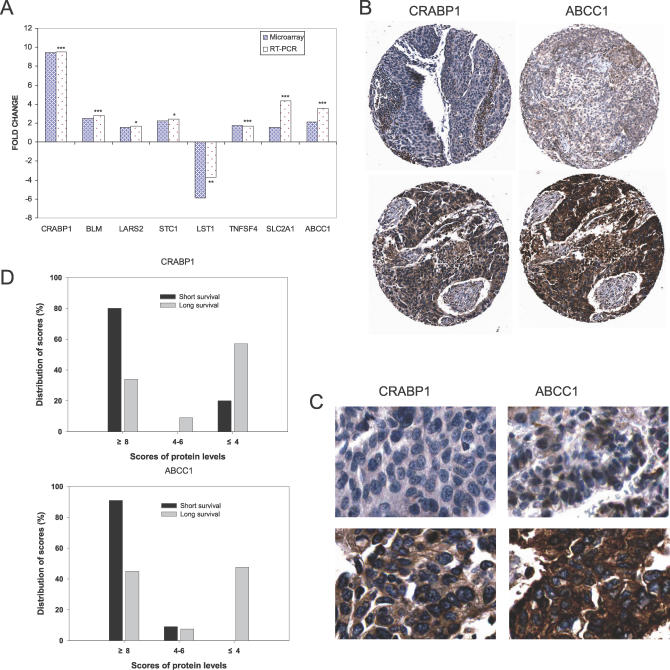
Validation Analyses of Gene Expression Profiling (A) QRT-PCR validations of several candidate survival-related genes. Bars represent fold changes for the selected genes with differential expression between long- (>5 y) and short-term survival (<2 y) patients. Positive fold change represents up-regulated, and negative fold change represents down-regulated in short-term survival patients. * *p* ≤ 0.05; ** *p* ≤ 0.01; *** *p* ≤ 0.005. (B and C) Immunostaining analysis of CRABP1 and ABCC1 expression in long- and short- term survival lung cancer patients. Low magnification (B) and 40× (C). Positive CRABP1 immunoreactivity was observed in cytoplasm of an acinar ADC (lower left photomicrographs of B and C) from short-term survival patients, and no CRABP1 reactivity was seen in a lung ADC from a long-term survival patient (upper left). Strong ABCC1 membranous staining (lower right) in tumor cells from short-term survival patients was observed, and weak ABCC1 reactivity was seen in a lung ADC from a long-term survival patient (upper right). (D) Distribution of CRABP1 and ABCC1 protein levels in short- and long-term survival patients.

To validate the microarray gene expression results from the meta-analysis, the relative expression of nine genes associated with overall survival *(CRABP1, BLM, ABCC1, SLC2A1, TNFSF4, BCL2, LST1, STC1,* and *LARS2)* was determined by QRT-PCR analysis on the samples from dataset 1. We confirmed the expression results for all these nine genes except *BCL2* (*p* ≤ 0.05) (Figure 1A).

The patient tissue microarray of 60 completely independent patients was interrogated for *CRABP1* and *ABCC1* to determine if mRNA changes were correlated with increased protein expression in lung ADCs from patients with short-term survival. CRABP1 staining was observed in the cytoplasm of tumor cells in most lung tumor tissues (Figure 1B and 1C). CRABP1 exhibited stronger staining in tissues of short-term survival patients than in those of long-term survival patients (Figures 1B, 1C, and S3), the scores for short- and long-term survival were 8.8 ± 3.1(mean ± SD, same hereafter) and 4.9 ± 2.8 (*p* < 0.0001), respectively. In short-term survival patients, 80% and 20% of the samples had scores of 8 or higher and 4 or lower in CRABP1 immunostaining, respectively; in contrast, in long-term survival patients, 34% and 59% of the samples had scores of 8 or higher and 4 or lower, respectively (Figure 1D). Similar trends were also observed in mRNA levels in our samples (see Figure 1A). ABCC1 showed either membranous or cytoplasmic staining in tumor cells of tissues of both short- and long-term survival NSCLC patients (Figures 1B, 1C, and S3). A significant increase in scores of ABCC1-positive staining was also present in tissues of short-term survival patients; the scores in tissues of short-term and long-term survival patients were 10 ± 2.3 and 6.6 ± 3.1 (*p =* 0.002), respectively. In short-term survival patients, 91% and 9% of the samples had ABCC1 immunostaining scores of 8 or higher and 4 or lower, respectively; in long-term survival patients, 45% and 48% of the samples had scores 8 or higher and 4 or lower, respectively. The results indicate that expression of these two proteins is consistent with the results from both microarray and RT-PCR analyses. Higher protein levels of CRABP1 and ABCC1 tend to increase risk of short survival of stage I NSCLC patients.

### Identification of a Gene Expression Signature for Survival

**Figure 2:**
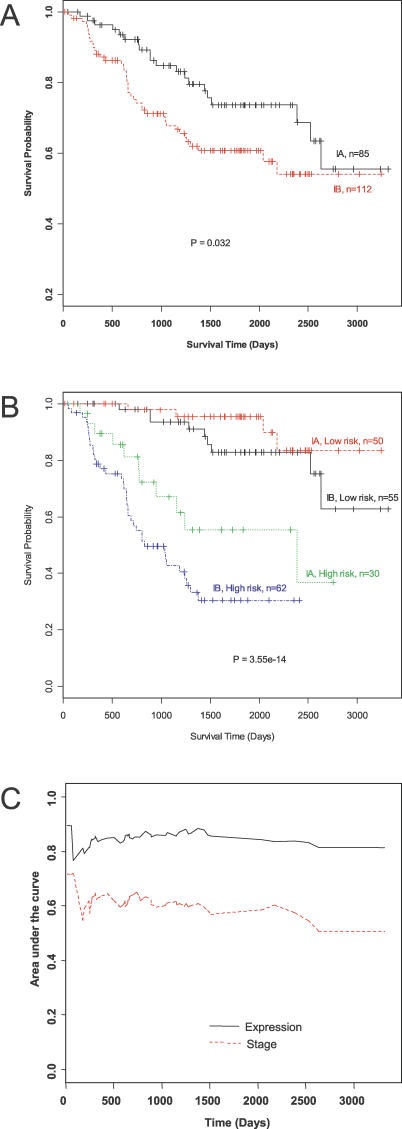
Survival Analyses of Stage I NSCLC (A) Kaplan-Meier survival curves for patients with stage IA and with IB NSCLC. (B) Kaplan-Meier survival curves for stage IA and IB patients defined by having positive (high-risk) or negative (low-risk) risk scores of overall survival. The risk scores were estimated with seven principle components based on the model built by 64 survival-related genes identified in five datasets. (C) Area under the ROC curve for survival models based on stage information or expression data, respectively.

Next, we determined if a subset of the genes related to overall survival can be used to predict survival of patients with stage I NSCLC. Risk scores were derived from survival analyses of all 197 samples in datasets 1 to 5 with the partial Cox regression. Kaplan-Meier survival analyses were performed after the samples were classified into high- and low-risk groups according to the risk scores. As shown in Figure 2A, Kaplan-Meier survival curves indicated poorer survival in stage IB than in stage IA NSCLC (*p* = 0.032). To determine whether gene expression profiles could accurately predict overall survival, the risk scores calculated by the 64 genes (listed in Table 2) were used to classify all of the samples from datasets 1 to 5 into two groups as high and low risk groups. Kaplan-Meier analysis using expression profiles demonstrated that the high and low-risk groups were significantly different in their overall survival (*p* < 0.001) (Figure 2B). A comparison of Figure 2A and 2B clearly shows that the gene expression signature has higher classification power than the staging method. The former has a larger area between the two risk groups and a smaller *p*-value from the Mantel-Haenszel log rank test. Figure 2C shows the time-dependent area under the ROC curves based on the stage information or the estimated risk scores of the patients. We observed that the Cox model with gene expression data gave the better predictive performance with the areas under the ROC curve close to 80%. The Cox model with stage information, in contrast, resulted in areas under the curve below 60%.

**Figure 3:**
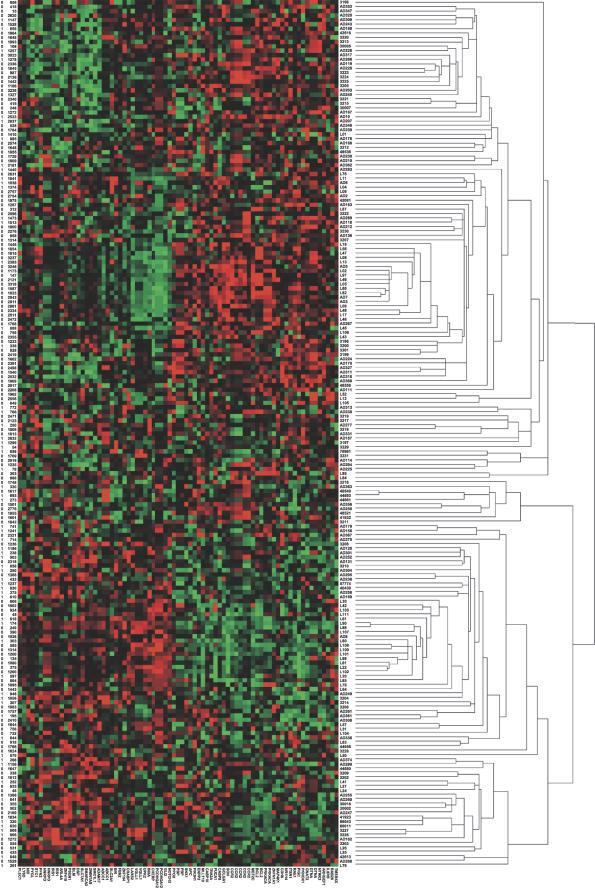
Gene Expression Patterns of 64 Top Survival Genes for 197 NSCLC Patients from Datasets 1 to 5 Patients were generally classified into two groups (short-term versus long-term survival) with distinct expression patterns. The first column on the left represents patient status: 0, alive; 1, dead; the second column on the left represents follow-up time (days).

Patients with a postoperative survival of at least 5 years and those who died within 2 years after resection were selected for estimating the predictive power of Kaplan-Meier survival analysis using expression profiles. According to the risk scores by the partial Cox regression approach, 77 of the 88 patients were classified correctly (87% accuracy) (Table S4). Gene expression patterns were determined using hierarchical clustering of the 197 NSCLC samples against the 64 top survival-related genes (Figure 3). Short- and long-term survival NSCLC patients had distinct expression patterns among the 64 genes that were used for establishing a gene expression signature predictive of stage I NSCLC survival.

### Confirmation of the Gene Expression Signature in Independent Datasets

**Figure 4:**
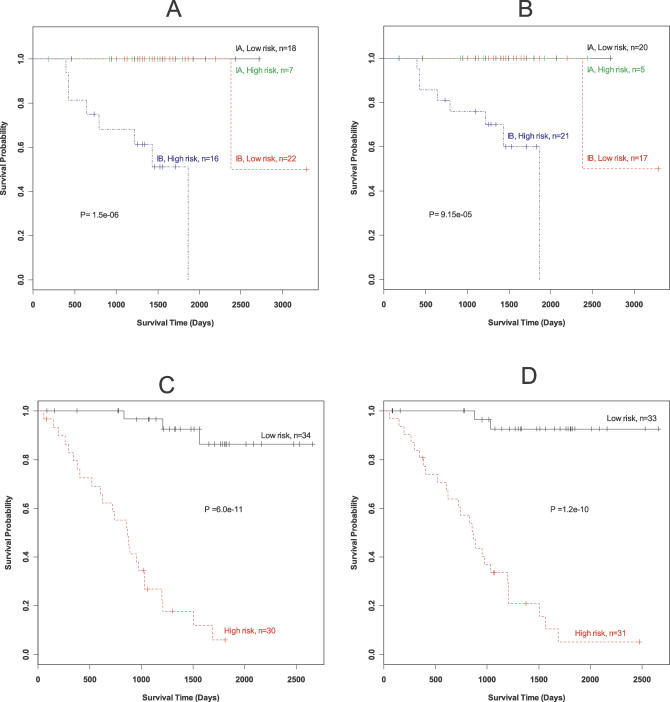
Comparison of the Prediction Accuracy of Lung Cancer Survival Using Our 64-Gene Signature and a Different 50-Gene Signature (A and B) Kaplan-Meier survival curves for dataset 6 under our 64-gene signature (A) and the 50-gene signature from Beer et al. [5] (B). Scores were estimated using two principle components. (C and D) Kaplan-Meier survival curves for dataset 7 using our 64-gene signature (C) and the 50-gene signature from Beer et al. [5] (D). Scores were estimated using eight principle components.

The robustness of the 64-gene expression signature in predicting survival in lung cancer was further tested with oligonucleotide gene expression data obtained from two completely independent datasets—dataset 6 (63 stage I lung ADC including nine long-term survival patients and five short-term survivors) and dataset 7 (64 stage I lung ADC and SCC, including eight long-term survival patients and twelve short-term survivors). When we examined the risk assignment of the samples in these two datasets using the risk scores based on our 64-gene signature, high- and low-risk groups were observed that differed significantly in survival in datasets 6 and 7. One of 14 individuals was classified incorrectly (93% accuracy) using the 64-gene signature in dataset 6 (*p* < 0.001; Figure 4A). In dataset 7, we correctly classified all 20 patients who survived for at least 5 years or died within 2 years using the 64-gene signature (*p* < 0.001; Figure 4C). We also examined the risk assignment of the samples in these two datasets using the risk scores based on the 50-gene signature reported by Beer et al. [5]. Classification was less accurate with this gene signature: three patients who lived for more than 5 years in dataset 6 were classified into the low-risk group according the risk scores calculated by our gene signature, but all of these patients were classified into the high-risk group under the Beer et al. [5] gene signature (Figure 4A and 4B). Also, in dataset 7 one patient surviving for more than 2,476 days was classified into the high-risk group under the Beer et al. [5] gene signature (Figure 4D).

## Discussion

In this study, we combined several lung cancer gene expression studies based on Affymetrix microarray platforms into a single, homogeneous dataset by using the DWD method. The increased sample size was intended to reduce false positives and increase statistical power in detecting survival-related genes. This improved dataset enabled us to identify a gene expression signature consisting of 64 genes that can accurately predict which stage I lung cancer patients may experience poor survival following resection.

In general, the pathologic diagnosis used to classify a lung tumor is combined with the stage of the cancer to predict patient survival and direct therapy [29]. Unfortunately, current methods of classification and staging are not completely reliable or sufficiently precise [2], and no reliable markers exist to predict the presence of micrometastasis or outcome in patients with resected NSCLC. It is not unusual for patients with lung cancers of identical histology, differentiation, location, and stage to have major differences in survival or response to therapy [29]. Some patients diagnosed with stage I NSCLC survive after surgery for some time, whereas others do not. This prognostic variability makes the results of this study important. Patients whose early-stage tumors contain signatures predicting short survival times would benefit from the aggressive therapies currently given only to those with later-stage cancers.

In this study, we included cancer subtypes as a factor in the ANOVA model to choose survival-related genes, and we adjusted cancer subtypes in the multivariate Cox proportional hazards regression analyses. Therefore, the gene expression signatures identified in the current study should be suitable for both lung ADC and SCC. This generalization of our gene signatures was also demonstrated in two independent large datasets—dataset 6 (63 stage I lung ADC including nine long-term and five short-term survival patients) and dataset 7 (64 stage I lung ADC and SCC, including eight long-term and 12 short-term survival patients). Our gene signatures can accurately predict patient survivals in these two datasets with mixed stage I lung cancer subtypes. To our knowledge, such signatures have not been convincingly reported previously, and we propose that they should be used to inform the clinical management of lung cancer patients.

Our survival gene signatures consist of genes that are involved in cancer metastasis such as cell adhesion *(APC, CDH8, DSP, LY6D, PCDHGA12,* and *NID),* cell motility *(IL8RB, ENPP2,* and *CCR2),* and inflammation and immune response *(CASP8* and *CASP10)*. In addition, seven of the genes are related to apoptosis *(INHA, PSEN1, CASP8, CASP10, PIK3R1, BCL2,* and *BIK)* and another five are related to transport mechanisms *(ABCC1, ITSN1, CRABP1, SLC2A1,* and *ZWINTAS)*. Nine of the signature genes were previously identified as lung cancer survival factors (Table 3), and 29 genes have been associated with survival in other cancer types including breast carcinoma, brain cancer, and gastric cancer (Table 4).

**Table 3:**
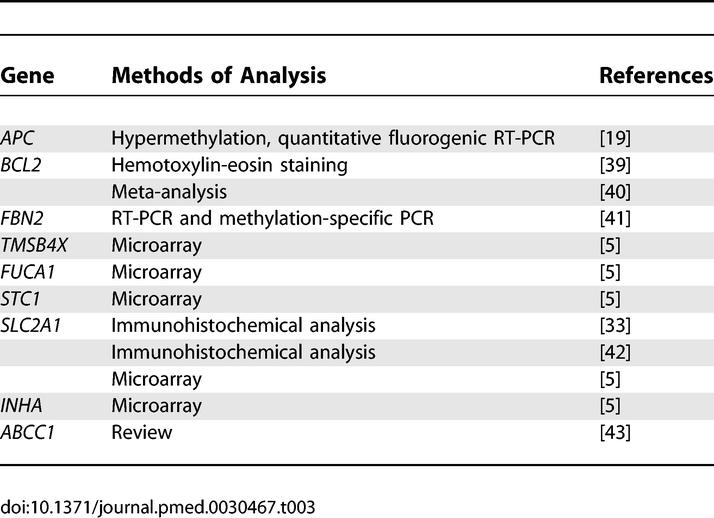
The Signature Genes of Survival Identified in Our Meta-Analysis Overlap Those in Previous Studies of Lung Cancer Survival

**Table 4:**
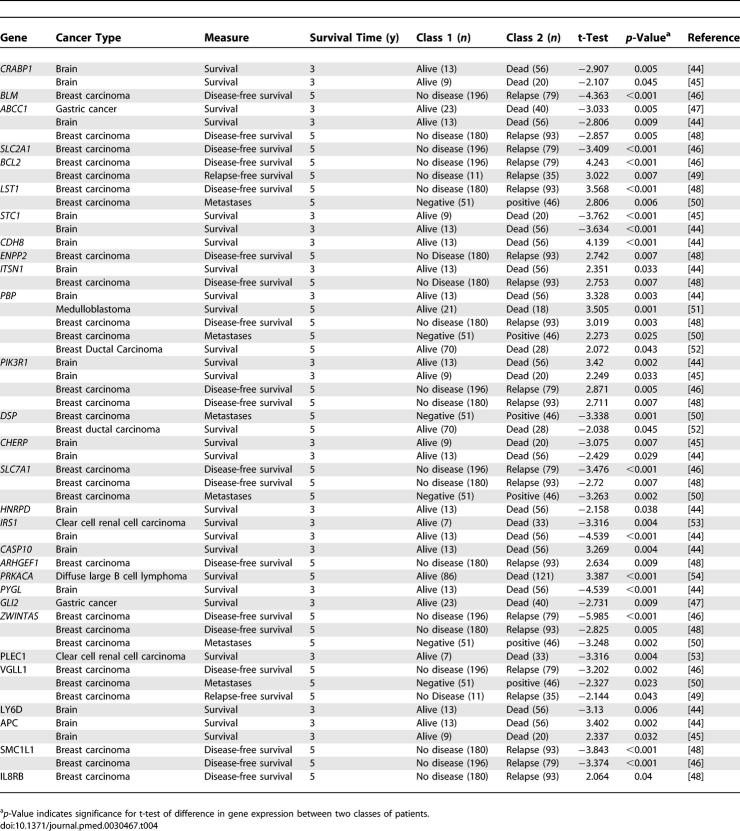
The Signature Genes of Survival Identified in Our Meta-Analysis Are Also Involved in Survival of Other Cancer Types

*ABCC1* and *SLC2A1* are particularly attractive biomarkers for survival in NSCLC. The protein encoded by *ABCC1* is a member of the superfamily of ATP-binding cassette transporters. ATP-binding cassette proteins transport various molecules across extra- and intracellular membranes. This full transporter is a member of the multidrug resistance-associated protein subfamily, and it functions as a multispecific organic anion transporter, with oxidized glutathione, cysteinyl leukotrienes, and activated aflatoxin B1 as substrates. This protein also transports glucuronides and sulfate conjugates of steroid hormones and bile salts. ABCC1 overexpression is associated with DNA aneuploid carcinomatous cells in NSCLC [30]. SLC2A1 is a major glucose transporter, which is an integral membrane glycoprotein involved in transporting glucose into most cells. Increased glucose transport in malignant cells has been associated with increased, deregulated expression of glucose transporter proteins that is characterized by the overexpression of SLC2A1 [31]. Differential expression levels of SLC2A1 have been observed between ADC and SCC [32], and overexpression of SLC2A1 in stage I NSCLC resulted in poor survival in another experiment [33]. Thus, these two genes could be targets of cancer therapy and prevention.

The survival-related genes identified in previous microarray studies of lung cancer patients failed to show consistency between studies, likely due to small patient sample numbers [34], and their predictive power was limited when tested in independent datasets. One solution to this problem is to integrate datasets from multiple studies to increase the sample size. Another problem is systematic biases due to different handling procedures in clinical studies, especially when samples/tumors are collected and processed at different institutions, using different microarray print batches, platforms, or array hybridization protocols. To integrate microarray datasets with different origins, distribution transformation methods, such as DWD, can be helpful. This method has been used previously to combine datasets from different batches into a single homogeneous dataset in head and neck SCC and breast carcinoma studies [35–38]. In our data analyses, we chose WUSM dataset as the reference batch, used the same mean and variance as reference, and then combined other datasets one by one. The hierarchical cluster analysis using the original nontransformed data classified the samples into five distinct groups according to data source rather than disease status (Figure S1A), demonstrating large systematic biases among the five studies. After DWD adjustment, however, all 197 samples from the five datasets were clustered into two sub-branches according to disease status rather than data source, each of which was composed of samples from all five datasets (Figure S1B). The batch differences disappeared; in this sense the samples from different datasets mixed well. Figure S4 also shows the effect of DWD adjustment using datasets 1 and 3. Principal component directions of adjusted data are markedly different from those of the raw data. We also performed poolability tests to examine if these DWD-transformed gene expression data from different resources are poolable. The results showed that the number of *p*-values falling in the tail in our data is similar to those in simulated data (Table S5). Therefore, the gene expression data from different resources are poolable after the DWD transformation, and thus can be combined for survival analysis. These results imply that the systematic biases were largely removed after DWD adjustment and thus the results from the integrated data should be robust.

Time to death due to cancer varies substantially among lung cancer patients. Studying censored survival time may be more informative than treating it as a binary or categorical variable. We applied multivariate Cox proportional hazards models with bootstrap resampling technology to the analysis of these censored survival data from different resource. Kaplan-Meier analysis using gene expression profiles demonstrated a significantly worse overall survival for high-risk patients compared to low-risk patients (Figure 2B), and using the 64-gene signature, we predicted the actual overall survival with greater than 85% accuracy. This new tool will help clinicians assess a patient's risk profile and to prescribe a course of treatment tailored to that profile. A patient whose cancer signature indicates that it is unlikely to metastasize would be spared the debilitating side effects of aggressive anticancer therapies, whereas a patient with an early but particularly aggressive tumor would be a candidate for aggressive treatment not usually given to early-stage patients, and thus experience improved survival.

## Supporting Information

Figure S1 Hierarchical Clustering Analysis of Five Datasets Analyses are shown for the raw data (A) and the DWD source and batch-adjusted data (B). Green, dataset 1 (WUSM); purple, dataset 2 (Mayo Clinic); blue, dataset 3 (Beer et al. [5]); red, dataset 4 (Bhattacharjee et al. [10]); yellow, dataset 5 (Borczuk et al. [8]).
(65 KB PDF)

Figure S2 Global *p*-Values from Tests of the Proportional Hazards Assumption for All Survival-Related Genes Only two of 165 tests obtained global *p* < 0.05, indicating that proportional hazards models are statistically warranted for the survival analyses.
(72 KB PDF)

Figure S3 The Immunostaining Images of Lung Cancer Patient Tissue Microarray The sections from short-term survival lung cancer patients are shown in box.
(1.3 MB PDF)

Figure S4 Principle Component Directions Directions are given for the raw data (A) and the DWD source- and batch-adjusted data (B). Red, dataset 1 (WUSM); blue, dataset 3 (Beer et al. [5]).
(513 KB PDF)

Protocol S1 Detailed Description of the Data Analyses (84 KB DOC)

Table S1 Sample Information on Datasets Used in the Meta-Analysis (73 KB XLS)

Table S2 Oligonucleotide Primers and Probes Used for RT-PCR Analysis (23 KB XLS)

Table S3 Detailed Information on Genes Related to Cancer Survival (32 KB XLS)

Table S4 Partial Cox Regression Classification of 197 stage I NSCLC patient using 64 Survival-Related Genes (32 KB XLS)

Table S5 Comparison of the Distribution of *p*-Values from Poolability Tests in the Real and Simulated Data (15 KB XLS)

### Accession Numbers

Accession numbers for genes related to cancer survival in Table S3 can be found in Nucleotide (http://www.ncbi.nlm.nih.gov/entrez/query.fcgi?db=Nucleotide). The Gene Expression Omnibus (GEO [http://www.ncbi.nlm.nih.gov/geo]) accession number for microarray data from this study is GSE6253.

## Abbreviations

ADC: adenocarcinoma
DWD: distance-weighted discrimination
NSCLC: non-small cell lung cancer
QRT-PCR: quantitative real-time polymerase chain reaction
SCC: squamous cell carcinoma
