# Supplementary material for: A Gene Expression Signature Predicts Survival of Patients with Stage I Non-Small Cell Lung Cancer
Source: PLoS Med. 2006 Dec 26;3(12):e467. doi: 10.1371/journal.pmed.0030467 (PMC1716187; doi:10.1371/journal.pmed.0030467)

Supplemental Figure 1

Hierarchical clustering analysis of 5 datasets for (A) the raw data and (B) the DWD source and batch adjusted data. Green: dataset 1 from WSU; Purple: dataset 2 from Mayo Clinic; Blue: dataset 3 from Beer et al. (2001); Red: dataset 4 from Bhattacharjee et al. (2001); Yellow: dataset 5 from Borczuk et al. (2004).

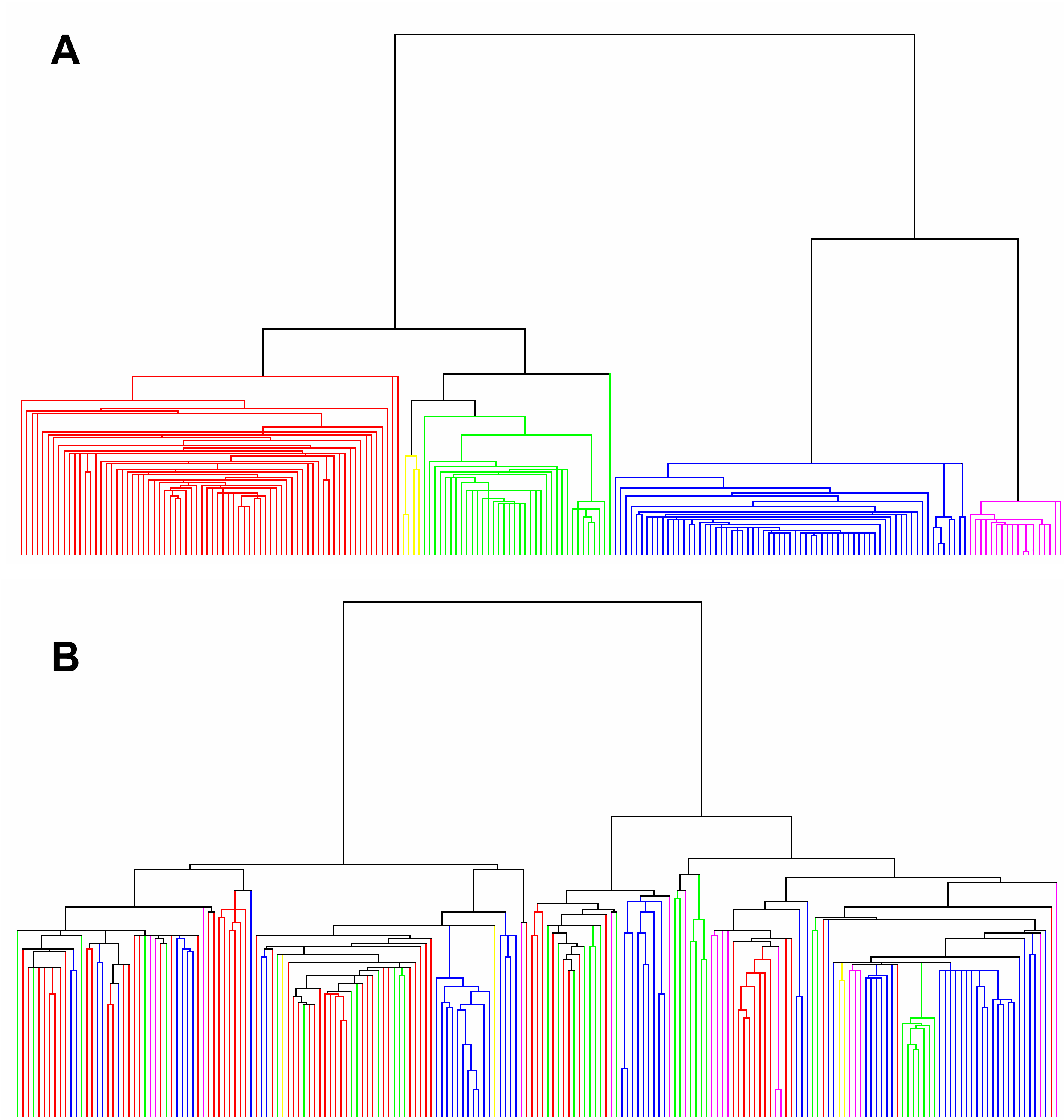

Supplement: Supplementary file 1 [file pmed.0030467.sg001.pdf]
