# Supplementary material for: A Gene Expression Signature Predicts Survival of Patients with Stage I Non-Small Cell Lung Cancer
Source: PLoS Med. 2006 Dec 26;3(12):e467. doi: 10.1371/journal.pmed.0030467 (PMC1716187; doi:10.1371/journal.pmed.0030467)

Figure S2

Global P values from testing the proportional hazards assumption for all the survival-related genes.

Only two out of 165 tests obtained global P < 0.05, indicating that the proportional hazards models are statistically warranted for the survival analyses.

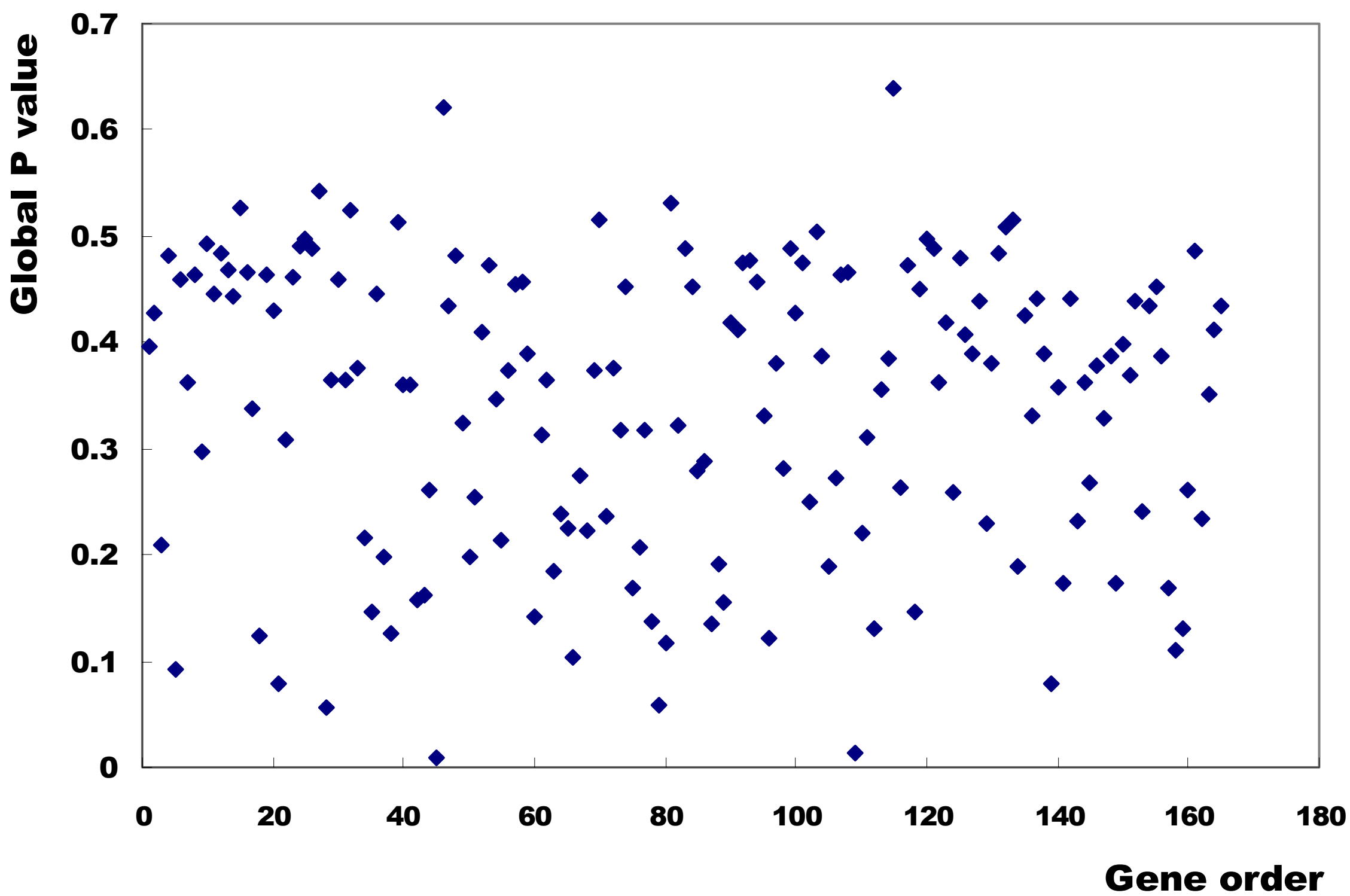

Supplement: Supplementary file 2 [file pmed.0030467.sg002.pdf]
