# Supplementary material for: A Gene Expression Signature Predicts Survival of Patients with Stage I Non-Small Cell Lung Cancer
Source: PLoS Med. 2006 Dec 26;3(12):e467. doi: 10.1371/journal.pmed.0030467 (PMC1716187; doi:10.1371/journal.pmed.0030467)

Supplemental Figure 3

The immunostaining images of lung cancer patient tissue microarray. The sections from short-term survival lung cancer patients are shown in box.

CRABP1

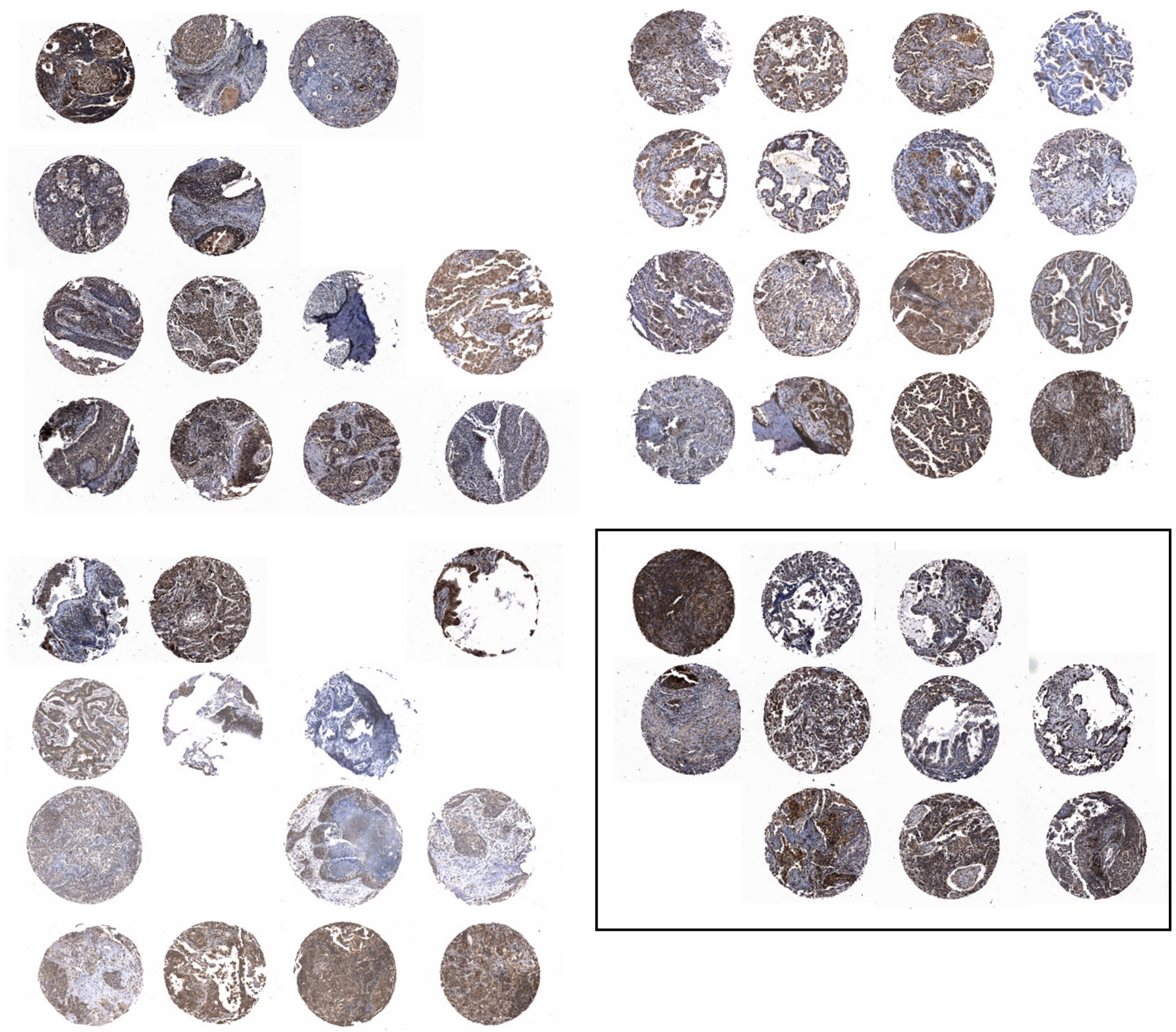

ABCC1

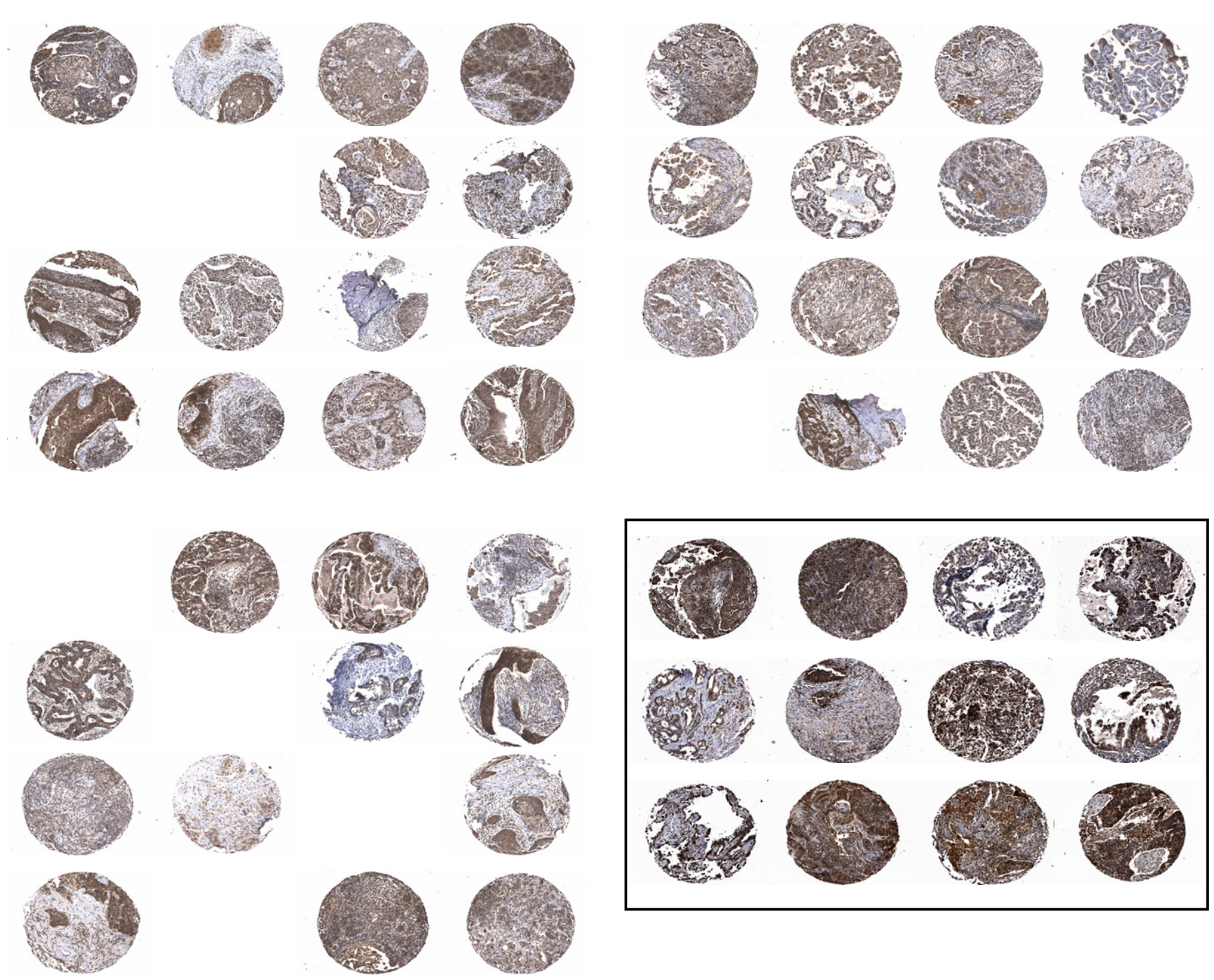

Supplement: Supplementary file 3 [file pmed.0030467.sg003.pdf]
