# Supplementary material for: A Gene Expression Signature Predicts Survival of Patients with Stage I Non-Small Cell Lung Cancer
Source: PLoS Med. 2006 Dec 26;3(12):e467. doi: 10.1371/journal.pmed.0030467 (PMC1716187; doi:10.1371/journal.pmed.0030467)

Figure S4

Principle component directions

Directions are given for the raw data (A) and the DWD source- and batch-adjusted data (B). Red, dataset 1 (WUSM); blue, dataset 3 (Beer et al. [5]).

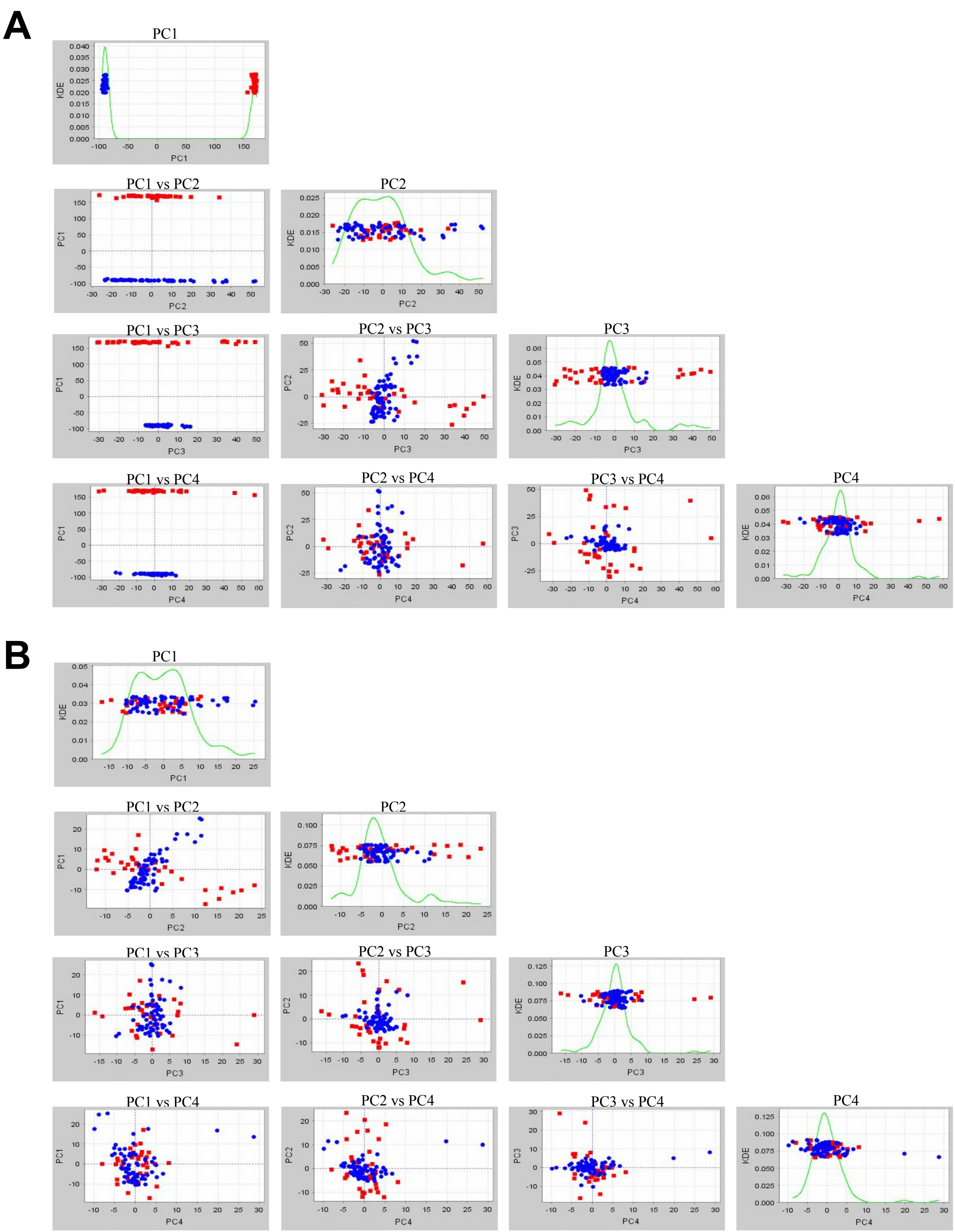

Supplement: Supplementary file 4 [file pmed.0030467.sg004.pdf]
