# Supplementary material for: A Gene Expression Signature Predicts Survival of Patients with Stage I Non-Small Cell Lung Cancer
Source: PLoS Med. 2006 Dec 26;3(12):e467. doi: 10.1371/journal.pmed.0030467 (PMC1716187; doi:10.1371/journal.pmed.0030467)
Supplement: Supplementary file 5 [file pmed.0030467.sd001.doc]

**Data Analysis**

***Preselection of survival-related genes***

*Step 1* ANOVA analysis. In datasets 1 to 5, there were 88 patients died within two years or survived beyond 5 years after surgery. These samples were used for identification of genes related to survival time (less than 2 years and greater than 5 years). The following ANOVA model was performed. Let $y_{\text{ijkl}(n)}$ be the DWD-adjusted gene expression level of gene $n$ from the $i$th cancer subtype, the $j$th cancer stage, the $k$th survival status and the $l$th sample. The gene expression level $y_{\text{ijkl}(n)}$ can be expressed as,

$$y_{\text{ijkl}(n)}=\mu_{(n)}+C_{i(n)}+T_{j(n)}+S_{k(n)}+\text{CT}_{\text{ij}(n)}+\text{TS}_{\text{jk}(n)}+\text{CS}_{\text{ik}(n)}+\text{CTS}_{\text{ijk}(n)}+\varepsilon_{\text{ijkl}(n)}$$

where $i=1$or 2, represents cancer subtype ADC or SCC; $j=1$or 2, represents cancer stage IA or IB; $k=1$or 2, represents survival time less than 2 years or greater than 5 years. The gene effect $\mu_{(n)}$captures the overall mean expression level of gene $n$ across the cancer subtypes, stages and survival time. The $C_{i(n)}$accounts for gene-specific subtype effect representing overall expression differences between two subtypes. The $T_{j(n)}$ accounts for stage effect that captures overall expression difference between stage IA and IB. The $S_{k(n)}$ captures overall expression difference between those with survival less than 2 years and those greater than 5 years. The terms$\text{CT}_{\text{ij}(n)}$, $\text{TS}_{\text{jk}(n)}$ and $\text{CS}_{\text{ik}(n)}$account for the second-order interactions between cancer subtypes, stages and survival. The term$\text{CTS}_{\text{ijk}(n)}$accounts for their third-order interaction, and $\varepsilon_{\text{ijkl}(n)}$ represents random errors. For each gene, we performed 10,000 permutation tests and obtained empirical p-values for each variance component. Genes with significant survival effects (P<0.01) were selected for Step 2.

*Step 2 Cox* proportional hazards regression analyses. Multivariate Cox proportional hazards regression analyses (adjusted for age, gender, cancer subtype and cancer stage) with 10000 bootstrap resampling approach were performed for each survival-related gene using all of 197 samples in datasets 1 to 5. The proportional hazards assumption for these variables were investigated by examining the scaled Schoenfeld residuals. Gender and cancer stage displayed significant deviation from the proportional hazards assumption, therefore, these two variables were taken as strata and others were covariate in our Cox proportional hazards model. The genes were ranked according to the bootstrap frequencies of P<0.01 for its gene expression in regression models.

***Identification of gene signature to predict survival outcome***

The following survival analyses were based on all of 197 samples in datasets 1 to 5. Partial Cox regression method was performed to construct predictive components (Li and Gui, 2004). Then these components were used in the Cox model for building predictive models for cancer patient survival. The principle components were chosen in the model to maximize R^2^ explained by the model. The risk scores were calculated by$f(x)=\sum_{j=1}^{G} \beta_{j}(X_{j}-\bar{x}_{\text{.}j})$, where $G$ represents the number of genes; $\beta_{j}$ represents the estimated coefficient of the $j$th gene; $X_{j}$represent gene expression levels of the $j$th gene in all the samples, $\bar{x}_{\text{.}j}=1/n\sum x_{\text{ij}}$; $n$ is sample size and $x_{\text{ij}}$is the gene expression level of gene $j$ from sample $i$. All the samples were classified into high and low risk groups according to the risk scores. Patients with risk scores less than zero potentially have long-term survivals and those larger than zero have short-term survivals after surgical resection. To choose an appropriate subset of genes for signature, we carried out a forward selection procedure: 1) increase one gene each time based on the rank of genes that were identified in the section of *Preselection of survival-related genes*; 2) perform the partial Cox regression analysis as described above and obtain the prediction accuracy using the chosen subset of genes; and 3) repeat steps 1 and 2 until the prediction accuracy is maximized.

To evaluate the predictive performance of the proposed survival gene signatures, we employed time-dependent receiver operating characteristic (ROC) analysis for censored data and area under the curve (AUC) as our criteria to assess survival predictions. The time-dependent sensitivity and specificity functions are defined as: $\text{sensitivity}(c,t)=P\left\{ X>c|D(t)=1 \right\}$ and$\text{specificity}(c,t)=P\left\{ X>c|D(t)=0 \right\}$. The corresponding ROC(t) curve for any time t is defined as the plot of {sensitivity(c, t)} versus {1 – specificity(c, t)}, with cutoff point c varying. X is the covariate and D(t) is the event indicator (here, death) at time t. The area under the curve, AUC(t), is defined as the area under the ROC(t) curve. A nearest neighbor estimator for the bivariate distribution function is used for estimating these conditional probabilities accounting for possible censoring (Akritas MG, 1994). AUC can be used as an accuracy measure of the diagnostic marker; the larger the AUC, the better the prediction model. AUC = 0.5 indicates no predictive power, whereas AUC = 1 represents perfect predictive performance.

Hierarchical clustering based on centered Pearson correlation coefficient algorithm and average linkage method was used to show the expression patterns of survival-related genes in datasets 1 to 5.

Kaplan-Meier survival analyses were implemented after the samples were classified into two risk groups. Differences of the survival risk between the two risk groups were assessed using the Mantel-Haenszel log rank test. The larger area between the two risk groups and its associated smaller p value from the Mantel-Haenszel log rank test implicate a better classification model.

All of the data analyses were implemented using the R statistical package.

**Reference**

Li H, Gui J (2004) Partial Cox regression analysis for high-dimensional microarray gene expression data. Bioinformatics 20 Suppl 1: I208-I215.

Akritas MG (1994) Nearest neighbor estimation of a bivariate distribution under random censoring. Annu Stat 22:1299–327.
